# Gene Expression Analysis Reveals Novel Gene Signatures Between Young and Old Adults in Human Prefrontal Cortex

**DOI:** 10.3389/fnagi.2018.00259

**Published:** 2018-08-27

**Authors:** Yang Hu, Junping Pan, Yirong Xin, Xiangnan Mi, Jiahui Wang, Qin Gao, Huanmin Luo

**Affiliations:** ^1^Department of Pharmacology, School of Medicine, Jinan University, Guangzhou, China; ^2^Department of Pathology and Pathophysiology, School of Medicine, Jinan University, Guangzhou, China; ^3^Institute of Brain Sciences, Jinan University, Guangzhou, China

**Keywords:** normal brain aging, prefrontal cortical regions, transcriptomic, weighted gene correlation network analysis (WGCNA), hub gene

## Abstract

Human neurons function over an entire lifetime, yet the molecular mechanisms which perform their functions and protecting against neurodegenerative disease during aging are still elusive. Here, we conducted a systematic study on the human brain aging by using the weighted gene correlation network analysis (WGCNA) method to identify meaningful modules or representative biomarkers for human brain aging. Significantly, 19 distinct gene modules were detected based on the dataset GSE53890; among them, six modules related to the feature of brain aging were highly preserved in diverse independent datasets. Interestingly, network feature analysis confirmed that the blue modules demonstrated a remarkably correlation with human brain aging progress. Besides, the top hub genes including PPP3CB, CAMSAP1, ACTR3B, and GNG3 were identified and characterized by high connectivity, module membership, or gene significance in the blue module. Furthermore, these genes were validated in mice of different ages. Mechanically, the potential regulators of blue module were investigated. These findings highlight an important role of the blue module and its affiliated genes in the control of normal brain aging, which may lead to potential therapeutic interventions for brain aging by targeting the hub genes.

## Introduction

Brain aging is characterized by a progressive loss of physiological integrity including loss of gray and white matter volume, a general loss of dendritic spines, loss of synaptic plasticity, increased axonal bouton turnover rates, and elevated inflammation, leading to impaired function and increased vulnerability to neurodegenerative disease (Salthouse, 2009; Dorszewska, 2013; Grillo et al., 2013; Lopez-Otin et al., 2013). However, the systematic cellular mechanisms behind the normal brain aging phenotypic changes in the absence of neurodegenerative disease of healthy older adults are only barely understood. Inspiringly, precision medicine has emerged as a new approach to health care base on the individual’s molecular drivers of disease (Montine and Montine, 2015). Therefore, applying this tailored and molecular mechanism-based approach to understand and reduce the negative impacts of brain aging are very promising. Even though recent reports have suggested the distinct changes in the expression of genes at the single neuron level (Kadakkuzha et al., 2013), the systematic cellular mechanisms behind the normal brain aging phenotypic changes in the healthy older adults are only barely understood. One method to study molecular mechanisms of aging is the high-throughput technology. However, the biased process in large changes analysis of differential gene expression, as well as lacking the consideration of the relationship between changing genes as a whole are inevitable drawbacks for this method (Furlong, 2013; Lou et al., 2017).

In order to explore the dynamic changes for understanding the system-level properties of normal brain aging progress in an unbiased manner, one network approach, named weighted gene correlation network analysis (WGCNA) is proposed. It can group functionally correlated genes into modules (Langfelder and Horvath, 2008). These modules are constructed by calculating a correlation network analysis of large, high-dimensional datasets, which are based on pairwise correlations between genes due to their similar expression profile, and can correlate with different stages of clinical traits (Langfelder and Horvath, 2008). The R package for WGCNA has been successfully applied in various biological contexts, e.g., cancer (Heiland D. et al., 2017; Sun et al., 2017), mouse genetics (Savas et al., 2017), and analysis of brain imaging data (Heiland D. H. et al., 2017), which can also be used to describe the correlation structure between gene expression profiles, image data, genetic marker data, proteomics data, and other high-dimensional data (Langfelder and Horvath, 2008). The R package along with its source code and additional material are freely available at https://cran.r-project.org/web/packages/WGCNA/WGCNA.pdf. Even though, WGCNA approach has provided a comprehensive characterization of the transcriptomic changes for disease’s functional interpretation and led to new insights into the molecular aspects of clinical-pathological factors, there are very few reports applying WGCNA to identify gene co-expression networks associated with normal brain aging. To fulfill this gap, we conduct a WGCNA method by calculating module-trait correlations based on GSE53890 public microarray dataset, which include 41 samples and 24,455 genes. This approach identifies six meaningful co-expression modules significantly related to normal brain aging and highly preserved in other brain aging datasets. Besides, hub genes contributing to normal brain aging are also verified. Herein, this paper is devoted to discovering novel gene signatures that greatly impact the progression of normal brain aging by WGCNA approach.

## Materials and Methods

### mRNA Expression Data

First, the microarray-based expression dataset GSE53890 provided by Lu et al. (2014) was downloaded from the NCBI Gene Expression Omnibus (GEO^1^). This dataset contained quantile normalized genome-wide expression profiles of adult human brain samples from prefrontal cortical regions, including samples from 12 young (<40 years), 9 middle aged (40–70 years), 16 normal aged (70–94 years), and 4 extremely aged (95–106 years). And these postmortem brain tissue samples used in this study were neuropathologically normal for age, and were derived from non-demented individuals (**Supplementary File 1, Table S16**). The dataset was produced using Affymetrix Human Genome U133 plus 2.0 arrays, which allowed the expression analysis of over 47,000 transcripts. The other microarray datasets referenced during the study (GSE1572, GSE71620, GSE30272, GSE21779, and GSE11882) were also available in the public repository from NCBI GEO datasets. All the other datasets supporting the findings of this study were available within the article and provided it as **Supplementary File 2, Table S1**. For the public datasets, its detailed experimental methods and descriptions could be found in the original references. Notably, only the human normal brain aging samples in these datasets were included in our study.

### Microarray Data Analysis

After the raw data of GSE53890 was downloaded in CEL format, it was pre-processed identically with the R package affy by using the Robust Multichip Average (RMA) function for background correction, normalization, and summarization with the quantiles method (Irizarry et al., 2003; Giulietti et al., 2016). For this purpose, a cross-platform common identifier, the array annotation data hgu133plus2.db was used to transform the array probes to the respective Entrez Gene ID. Probes matching multiple genes were removed from the dataset, and then we calculated the average expression values of genes matching multiple probes. A proper threshold was set based on the amount of genes filtered out.

### Gene Co-expression Network Construction

Co-expression networks were constructed using WGCNA (v1.47) package in R (Langfelder and Horvath, 2008). After filtering genes, gene expression values were imported into WGCNA to construct co-expression modules using the automatic network construction with default settings. First, a matrix of adjacencies using the WGCNA function adjacency was constructed by calculating Pearson correlations between all pairs of genes across all selected samples, after which this matrix was computed into a Topological Overlap Matrix (TOM) using the function TOMsimilarity (Zhang and Horvath, 2005). The TOM, referred to the interconnection between two genes, was used as input for hierarchical clustering analysis, and a cluster of genes with high topological overlap was defined as a module. Finally, modules were identified on the dendrogram with the function cutreeHybrid from the R package dynamicTreeCut algorithm (Langfelder et al., 2008). The module eigengene (ME) was considered as a representation of the gene expression profiles in a module, which was defined as the basic component of a given module (Langfelder and Horvath, 2008). The module membership (MM) was calculated by the WGCNA function signedKME that correlated the ME with gene expression values, so it quantified how close a gene was to a given module (Langfelder and Horvath, 2008). Moreover, genes, which were infirmly correlated with all of the MEs (|kME| < 0.7), were assigned to none of the modules (Lou et al., 2017). Finally, the interesting module network was visualized by Cytoscape_3.3.0 (Demchak et al., 2014).

### Calculation of Module-Trait Correlations and Module Preservation

Correlations among gene expression modules and phenotypic trait for GSE53890 were investigated; age and sex were chosen as our interesting trait. Modules having significant relationships with age trait were listed in **Supplementary File 1, Table S3**. Modules were labeled with a conventional color scheme. Besides, a WGCNA integrated function (modulePreservation) was applied to calculate module preservation statistics between two relevant datasets. And then, two composite preservation statistics for module preservation were delineated as follows: the definition of Zsummary was the average of *Z*-scores computed for density and connectivity measures, which represented the significance of observed statistics. Analogously to the definition of median rank, the statistic median rank was defined as the average calculation of median ranks for connectivity and density measures of each module (Langfelder et al., 2011; Lou et al., 2017). Eventually, median rank was useful for identifying relative preservation among multiple modules; if a module had a lower median rank, it tended to exhibit stronger observed preservation statistics than a higher one. Zsummary was used to assess the significance of observed statistics by distinguishing preserved from non-preserved modules via permutation testing 200 times (Langfelder et al., 2011; Lou et al., 2017).

### Feature Vectors in WGCNA Network

The correlation between individual genes and biological trait (age and sex) was defined as the gene significance (GS). The summation of adjacency performed over all genes in a particular network was calculated as the intramodular connectivity (K.in). Generally, if GS and MM were highly associated, it implied that genes were the highly important elements for modules and were most significantly correlated with the trait. Meanwhile, if the MM was highly related to K.in, it indicated that a gene was more vital than the given module (Zhang and Horvath, 2005; Lou et al., 2017). From above, hub genes were usually characterized with high GS, high MM and high K.in in a module, which were highly connected with other genes and hence of high functional significance, as well as tended to be located in the center of a module network (Lou et al., 2017).

### Functional Annotation of the Modules

For genes in each module, Gene Ontology (GO) and KEGG pathway enrichment analysis were conducted to analyze the biological functions of modules. Significantly enriched GO terms and pathways in genes in a module comparing to the background were defined by hypergeometric test and with a threshold of false discovery rate (FDR) less than 0.05. The Enrichr database^2^ contained a large collection of gene set library; these libraries had been constructed from many sources such as published studies and major biological and biomedical online databases (Chen et al., 2013; Giulietti et al., 2016). Thus, we input the interesting modules into the Enrichr by comparing them to the annotated gene sets libraries. Enrichr implemented four scores to assess enrichment results: *p*-value, *q*-value, rank (*Z*-score), and combined score. The rank score or *Z*-score was computed to assess the deviation from an expected rank by using a modification to Fisher’s exact test. Finally, the combined score was calculated by multiplying the two scores as follows: *C* = log(*p*)^∗^*Z*. Where *C* is the combined score, *p* is the *p*-value computed using Fisher’s exact test, and *Z* is the *Z*-score computed to assess the deviation from the expected rank (Chen et al., 2013; Giulietti et al., 2016; Lou et al., 2017).

### Animal Study and Histological Analysis of Mouse Brain

Animal housing and experiments were carried out according to the guidelines of the Animal Ethics Committees of Jinan University and were performed under the standard biosecurity and institutional safety procedures. Male C57BL/6J mice (3-month-old and 12-month-old) were maintained in a 12-h light–dark-cycle at room temperature with access to food and water *ad libitum* in our animal facilities. The mice were divided into two groups (3-month-old and 12-months-old). At the end of the experiments, brains were fixed by intracardial perfusion with 4% (vol/vol) paraformaldehyde (PFA) in PBS, followed by the fixation in the same mixture overnight. Then, they were processed for paraffin embedding, according to standard procedures. A part of the brain tissue was homogenated in TRIzol^®^ Plus RNA Purification Kit (Life Technologies), and one microgram of RNA was then reverse transcribed to cDNA using the High Capacity cDNA Reverse Transcription Kit (Invitrogen) for the quantitative real-time PCR analysis. Formalin-fixed brain tissue was processed into 4 μm thick paraffin sections and stained with hematoxylin and eosin (HE) staining. For quantification of neuronal density, randomly selected areas within the hippocampus or the cortex were imaged at a magnification fluorescent microscope (Carl Zeiss, Axio Imager.A2, Germany).

### MDA and SOD Determination

About 200 ± 50 mg brain tissues of prefrontal cortex (PFC) was taken and washed by precooled normal saline (NS) for at least three times. And then, they were converted to 100g/L of brain homogenates in a homogenizer filled with nine times the mass of precooled NS. The homogenates were centrifuged at 4°C for 20 min at a speed of 3500 r/min. The protein quantification of supernatant was estimated by BCA method. And then, proper amount (50–100 μg) of supernatant’s lipid peroxidation levels (MDA) and SOD activity were measured according to the specifications of MDA kit (S0131, Beyotime, China) and SOD kit (S0101, Beyotime, China).

### qPCR Analysis

Total RNA from PFC brain tissue was extracted by TRIzol (Invitrogen, United States). Synthesis of cDNA was performed by using 2 μg of total RNA with PrimeScript^TM^ Reverse Transcriptase (Takara) according to the manufacturer’s instructions. Specific primers used for PCR were listed as follow:

5′-GTAACCCGTTGAACCCCATT-3′ (18S rRNA-sense),

5′-CCATCCAATCGGTAGTAGCG-3′ (18S rRNA-anti-sense);

5′-CCTGAACACCGCACATAC-3′ (Ppp3cb-sense),

5′-CATCACCTTGGTCAACCC-3′ (Ppp3cb-anti-sense);

5′-GAAGGCCTGGCTTACCTACC-3′ (Camsap1-sense),

5′-AGACCCAAAGCAGCTACACC-3′ (Camsap1-anti-sense);

5′-CCAAAGGAGGGTGTTGAGAGG-3′ (Actr3b-sense),

5′-GCCATGTCGTATAGGCCACTT-3′ (Actr3b-anti-sense);

5′-GCACTATGAGTATTGGTCAAGCA-3′ (GNG3-sense),

5′-GTGGGCATCACAGTATGTCATC-3′ (GNG3-anti-sense).

The gel image was acquired in the Gel Doc 1000 system and analyzed using the Quantity One software (Bio-Rad Laboratories, Hercules, CA, United States). 18S rRNA was chosen as the endogenous control and cycle dependence was carried out to ensure that the PCR products fell within the linear range. Quantitative real-time PCR was performed using the SYBR^®^ Premix Ex Taq Kit (Takara) in a 7900 Real Time PCR System (Applied Biosystems, United States) for at least three independent experiments. The relative quantification expression of each gene was normalized to 18S rRNA, and calculated using the 2^-ΔΔΔCT^ method.

### Statistical Analysis

All experiments were performed for at least three independent times, and the data were expressed as the mean ± standard deviation (SD). All statistical analysis was performed using GraphPad Prism 6 Software (GraphPad Software, San Diego, CA, United States). Comparison between two groups was conducted by using Student’s *t*-test. *P*-values less than 0.05 were considered as statistically significant.

## Results

### Pre-processing of the Aging Human Prefrontal Cortex Datasets and Construction of Weighted Gene Co-expression Networks

**FIGURE 1:**
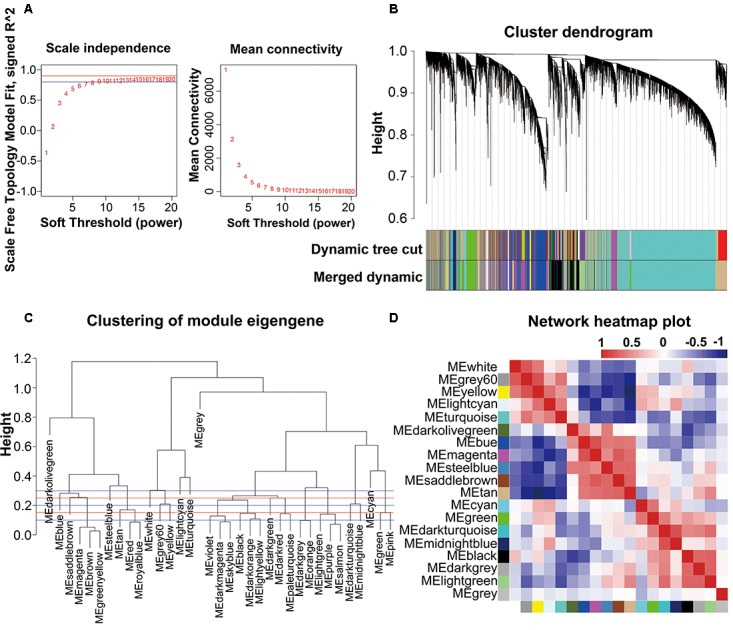
WGCNA network and module detection. **(A)** Selection of the soft-thresholding powers. The left panel showed the scale-free fit index versus soft-thresholding power. The right panel displayed the mean connectivity versus soft-thresholding power. Power 8 was chose, for which the fit index curve flattens out upon reaching a high value (>0.9). **(B)** Cluster dendrogram and module assignment for modules from WGCNA. Genes were clustered based on a dissimilarity measure (1-TOM). The branches correspond to modules of highly interconnected groups of genes. Colors in the horizontal bar represent the modules. 35 modules with 24,455 transcripts were detected with WGCNA. **(C,D)** Meta-module identification and module-module relationship. The module network dendrogram was constructed by clustering module eigengene distances. The horizontal line (blue and red line) represents the threshold (0.2) used for defining the meta-modules. Thus, 19 distinct gene modules were identified. In the heatmap of module-module relationship, the progressively more saturated blue and red colors indicated the high co-expression interconnectedness.

The combined dataset (GSE53890) containing a total of 41 samples [12 young (<40 years), 9 middle aged (40–70 years), 10 normal aged (70–90 years), and 10 extremely aged (90–106 years)] with clear brain aging staging was applied into this study (**Supplementary File 2, Table S1 and Figure S1**). Raw data from each microarray dataset were then pre-processed identically for background correction and normalization. Firstly, probes matching multiple genes were removed out from these datasets, and secondly the average expression value of gene measured by multiple probes was calculated as the final expression value. Finally, we identified in total 24,455 genes that were expressed (**Supplementary File 1, Table S1**). Besides, constructing a WGCNA needed an optimal soft-thresholding power to which co-expression similarity was raised to calculate adjacency. Thus, we performed the analysis of network topology for various soft-thresholding powers in order to have relative balanced scale independence and mean connectivity of the WGCNA. As shown in **Figures 1A,B**, power 8, the lowest power for which the scale-free topology fit index reached 0.90, was chosen to produce a hierarchical clustering tree (dendrogram). Next, through dynamic tree cut and merged dynamic, 35 distinct gene modules were generated in the hierarchical clustering tree (dendrogram) from 41 samples and each module labeled by different colors was shown by the dendrogram (**Figure 1B**), in which each tree branch constituted a module and each leaf in the branch is one gene. The size of modules ranged from 64 (darkolivegreen module) to 9,296 (turquoise module) genes. As shown in **Figure 1C**, the module network dendrogram was constructed by clustering ME distances. Modules with high K.in were located at the tip of the branches since they exhibit the highest interconnectedness with the rest of the module. The horizontal line (blue and red line) represented the threshold (0.2) used for defining the meta-modules. Thus, 19 distinct gene modules were identified. To further quantify co-expression similarity of entire modules, we calculated their eigengenes adjacency on their correlation of the entire modules (**Supplementary File 2, Figure S2**) and 19 modules (**Figure 1D**) based on the heatmap, respectively. Each module showed independent validation to each other as well, and the progressively more saturated blue and red colors indicated the high co-expression interconnectedness (**Figure 1D**). All attributes of genes and samples were shown in **Supplementary File 1, Tables S2, S3**.

### Identification of Meta-Modules Related to the Brain Aging

**FIGURE 2:**
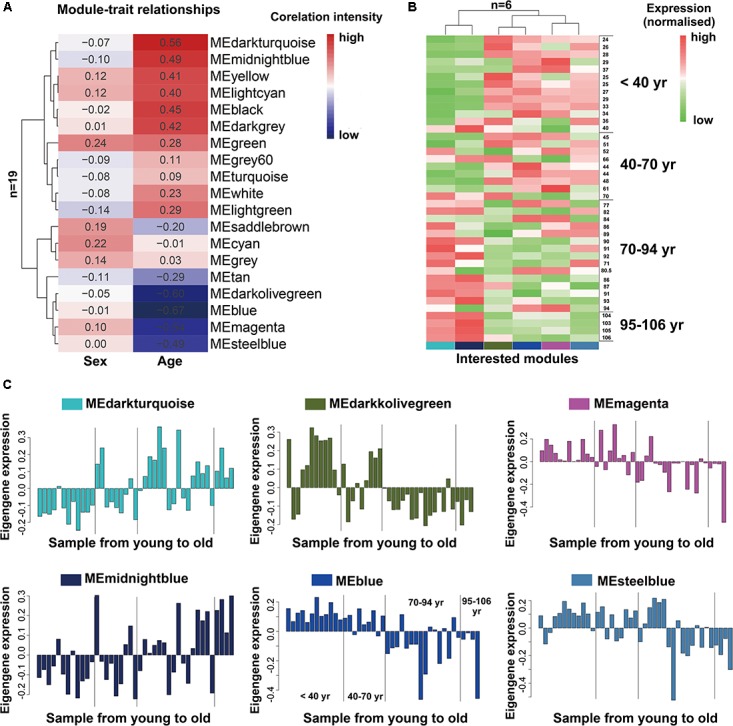
Module-trait and the gene expression of interested modules in the aging human prefrontal cortex. **(A)** Supervised hierarchical cluster of each row correspond to a module eigengene (*n* = 19), column to a trait. Each cell contained the corresponding correlation. High correlations was colored in red, low correlation in blue. **(B)** Hierarchical cluster analysis of six interested modules, based on the module-trait’s correlation and p value (absolute *r* > 0.5, *P* < 10–2), four modules (blue, darkolivegreen, magenta, steelbule) showed relatively high expression in young adults (red) and lower expression (green) in the aging population. Conversely, the other two modules (darkturquoise and midnightblue) showed the opposite result. Each lane represented an individual prefrontal cortical brain sample. **(C)** The histograms described the eigengene expression of each module from young to old.

As we known, the ME is the first principal component of a given module and can be considered as a representative of the module’s gene expression profile. The 19 MEs for the 19 distinct modules were each correlated with age trait, which has been shown in eigengenes trait-specific expression profiles (**Supplementary File 1, Table S4**). Next, we evaluated the relationship between each module and aging status by correlating the eigengenes of each module with age and sex traits. The age and sex traits include the whole age and sex range in 41 individuals (**Supplementary File 2, Table S2**). We found that, as expected, six modules (blue, darkolivegreen, darkturquoise, magenta, steelbule, midnightblue) exhibited similar characteristics in age trait (absolute *r* > 0.5, *P* < 10^-2^; **Figure 2A**), while others were not preserved. Notably, among them, four modules (blue, darkolivegreen, magenta, steelbule) were negatively correlated with age (*r* < -0.5, *P* < 10^-2^; **Figure 2A**), thereafter named anti-aging module. Two positively correlated modules (darkturquoise and midnightblue) named aging module thereafter. Besides, in our study, we found that the sex trait had no significant relationship with the 19 distinct modules, so we just simply ignored it. Further, a consensus clustering also confirmed the two main group were clearly separated by the 41 aging samples from young to old (**Figure 2B**). Similarly, the six interesting modules based on ME expression profile and 41 samples with extract age trait from young to old were also displayed in **Figure 2C**. The module eigengene E in *Y*-value was defined as the first principal component of a given module. It can be considered a representative of the gene expression profiles in a module. The *X*-value of **Figure 2C** from young to old in exact ages was shown in **Supplementary File 2, Table S2**. Modules were labeled using a conventional color scheme.

### Module Stability and Preservation Analysis

**FIGURE 3:**
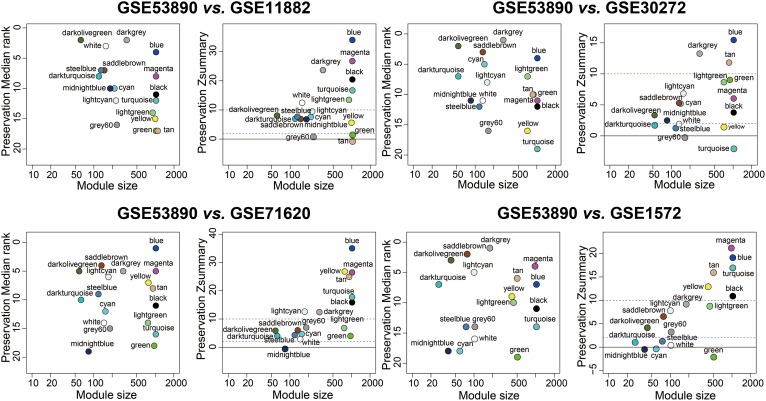
Preservation analysis of GSE53890 network modules in different brain aging datasets. Each module was represented by its color-code and name. Left figure showed the composite statistic preservation median rank. This measure tended to be independent from module size with high median ranks indicating low preservation. Right figure showed preservation Zsummary statistic. The dashed blue and green lines indicated the thresholds *Z* = 2 and *Z* = 10, respectively. Zsummary < 2 implied no evidence for module preservation, 2 < Zsummary < 10 implies weak to moderate evidence, and Zsummary > 10 implies strong evidence for module preservation. The anti-aging modules (blue, magenta, darkolivegreen) showed high preservation statistics summary than expected by random chance using bootstrapping validation procedures.

To test the stability of the indicated modules, a WGCNA integrated function (modulePreservation) was applied to calculate module preservation statistics and the Zsummary score (*Z*-score) was used to evaluate whether a module was conserved or not. Modules with a *Z*-score > 10 were regarded as highly preserved. To ascertain if the identified modules were preserved in other different datasets, an independent validation was carrying out. We retrieved four datasets, which was relevant to brain aging and all samples were from human PFC. Results showed that the anti-aging modules (blue, magenta, darkolivegreen) were preserved stably in GSE11882, GSE30272, GSE71620, and GSE1572 datasets (**Figure 3**), while aging modules (darkturquoise, midnightblue) showed weak to none evidence for module preservation according to the summary preservation analysis (**Figure 3**). The blue and magenta modules were regarded as the highly representative aging-associated modules, because they both made a higher conservation and consistent association with brain aging.

### Functional Enrichment Analysis of the Gene Modules of Interest

To explore the biological functions of the anti-aging modules (blue, magenta, darkolivegreen), we performed GO term enrichment analysis, as well as pathway ontology analyses by using the Database for Annotation, Visualization and Integrated Discovery (DAVID^3^) (Huang da et al., 2009). Top biological processes and KEGG pathway in each module was shown in **Table 1**. For the blue module, the top two enriched terms in GO ontology were “transport” (FDR = 3.34E-15) or “establishment of localization” (FDR = 3.34E-15). For the KEGG pathway analysis, the top enriched terms were “Synaptic vesicle cycle” (FDR = 1.07E-09) and “cGMP – PKG signaling pathway” (FDR = 3.14E-08). For magenta module genes, the top enriched terms in the GO and KEGG pathway databases were “mitochondrion organization” (FDR = 1.50E-39) and “Oxidative phosphorylation” (FDR = 2.98E-19). Moreover, genes in darkolivegreen module were found to be significantly enriched in cell–cell signaling of the GO term and circadian entrainment signaling pathway. The complete annotation for each module was provided in **Supplementary File 1, Tables S5, S6**. These findings together with previous research implied that extensive oxidative phosphorylation and accelerated mitochondrion organization were the fundamental characteristics of brain aging.

**Table 1:** Top GO and pathway enrichment in each module.

	Module	Category	Term	*P*-Value	FDR
Anti-aging module	Blue	GOTERM_BP	GO:0006810-transport; GO:0051234-establishment of localization	6.54E-17	3.34E-15
	Darkolivegreen	GOTERM_BP	GO:0007267-cell–cell signaling	1.69E-10	2.12E-08
	Magenta	GOTERM_BP	GO:0007005-mitochondrion organization	2.06E-41	1.50E-39
	Steelbule	GOTERM_BP	GO:0007267-cell–cell signaling	3.11E-03	4.86E-01
Aging module	Darkturquoise	GOTERM_BP	GO:0016070-RNA metabolic process	1.29E-02	3.38E-01
	Midnightblue	GOTERM_BP	GO:0090304-nucleic acid metabolic process	5.64E-02	9.83E-01
	**Module**	**Category**	**Term**	***P*-Value**	**FDR**
Anti-aging module	Blue	KEGG_PATHWAY	ko04721:Synaptic vesicle cycle	3.69E-12	1.07E-09
	Darkolivegreen	KEGG_PATHWAY	ko04713:Circadian entrainment	4.48E-03	1.76E-01
	Magenta	KEGG_PATHWAY	ko00190:Oxidative phosphorylation	1.09E-21	2.98E-19
	Steelbule	KEGG_PATHWAY	ko05032:Morphine addiction	7.74E-09	6.89E-07
Aging module	Darkturquoise	KEGG_PATHWAY	ko04914:Progesterone-mediated oocyte maturation	1.26E-02	3.13E-01
	Midnightblue	KEGG_PATHWAY	ko03022:Basal transcription factors	6.82E-04	4.70E-02

### Network Analysis of the Gene Modules of Interest

**FIGURE 4:**
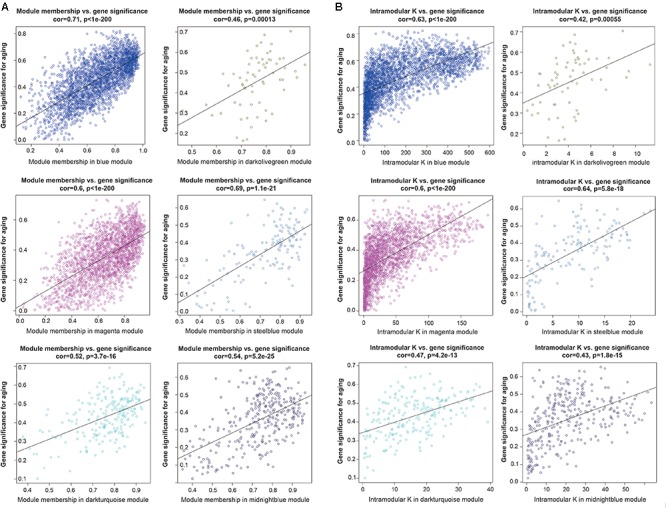
Module features of GS, MM and K.in. **(A)** Modules significantly correlated with aging status (young versus aged cases). Each point represented an individual gene within each module, which were plotted by GS on the *y*-axis and MM on the *x*-axis. The regression line, correlation value, and *p*-value were shown for each plot. **(B)** Correlation of the K.in (*x*-axis) and the GS (*y*-axis).

To further investigated the gene constitution of particular modules which were most related with the brain aging, three network unique properties such as GS, MM, and K.in were carried out. Abstractly speaking, if a gene is higher with GS, MM, and K.in, it is more meaningful with the clinical trait (Langfelder et al., 2013; Lou et al., 2017). Thus, a specific module whose MM, K.in or GS were significantly connected and associated with the brain aging trait; it implied that this module may play a more important biological role on aging progression (Lou et al., 2017). Of the six interesting modules, blue, magenta and steelblue modules showed significant correlations between MM and GS. Similarly, there were also a markedly correlation between GS and K.in in the blue, magenta, and steelblue modules (**Figure 4**). Overall, module blue were observed as the best meaningful module by its strongly positive correlations (*r* = 0.71, *p* < E-200 in GS vs. MM; *r* = 0.63, *p* < E-200 in GS vs. K.in). These results indicated that blue module was closely involved in human brain aging progression.

### Characterization of the Blue Module Content and Hub Genes

**FIGURE 5:**
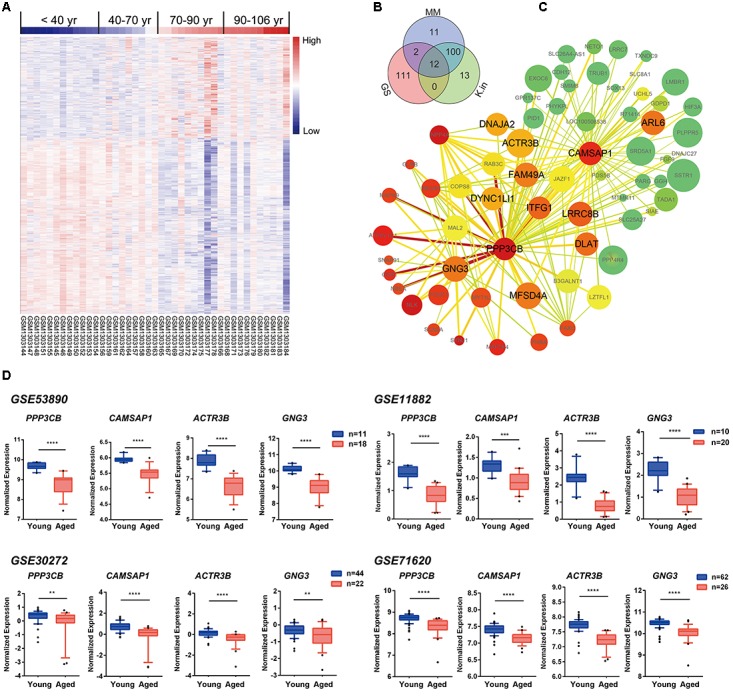
Characterization of the blue module. **(A)** Heat map showing hierarchical clustering of each samples based on the expression of the blue module genes. **(B)** Venn diagram of blue module genes in the top of 125 based on high gene significance (GS), high module membership (MM), and high intramodular connectivity (K.in). **(C)** Interaction of gene co-expression patterns in the blue module. The module was visualized using Cytoscape_3.3.0 software. The node colors coded from green to red (low to high) indicated the K.in level when compared young with advanced brain aging state. The node size was proportional to the GS with age trait. The higher of the GS, the bigger of the node size. **(D)** Four hub genes expression pattern in brain tissues according to GSE53890, GSE71620, GSE30272, and GSE11882 cohort. Data were shown as box and whisker plot. Student’s *t*-test was used for statistical analysis. ^∗∗^*p* < 0.0, ^∗∗∗^*p* < 0.001, ^∗∗∗∗^*p* < 0.0001.

To explore the blue module’s gene expression profiles and its distribution in the 41 samples, a hierarchical cluster analysis was carried out, and result showed higher expression in young adults (red) and lower expression (blue) in the 41 aging population (**Figure 5A**). In the following, we focused on the core genes of the blue module; the core genes usually characterized by a high GS for aging status, as well as high MM and K.in. Thus, network top interesting genes (top125) of the blue module based on the above three indexes were listed in the Venn diagram and 12 genes were the intersections (**Figure 5B** and **Supplementary File 1, Table S10**). Similarly, we modeled a network view of blue module by cytoscape with TOM ≥ 0.25 and the 12 hub genes of blue module was depicted in **Figure 5C** and **Supplementary File 1, Tables S11, S12**. The values of each gene in the network view of blue module based on the three parameters were as follow: The K.in count ranged from 74.34 to 595.30, with an average of 366.11 ± 115.46; The GS score ranged from -0.82 to 0.54, with an average of -0.59 ± 0.087; The MM count ranged from -0.91 to 0.98, with an average of 0.81 ± 0.35. Further, applying GeneMANIA18 database to simulate the blue network gave the similar results (**Supplementary File 2, Figure S4**). PPP3CB and CAMSAP1, based on MM and K.in indexes, were the two top network hub genes and another two top genes (ACTR3B and GNG3) ranked on GS were also disclosed. Specifically, gene microarray in animal study has identified in region CA3, the catalytic and regulatory subunits for the phosphatase calcineurin (PPP3CB) are up-regulated by caloric restriction influences (Zeier et al., 2011). The homologene of PPP3CB in *C. elegans*, tax6 (C02F4.2), has been reported to regulate *C. elegans*’ lifespan through DAF-16 (Tao et al., 2013), and it also has a multiple functions in its development, fertility, proliferation, and behavior (Lee et al., 2013). To the best of our knowledge, there has been nothing directly implicating CAMSAP1, ACTR3B, and GNG3 reported to be associated with aging. However, ACTR3B has been showed involved in age-associated cognitive dysfunction in the rat hippocampus (Ottis et al., 2013). CAMSAP1 (Calmodulin Regulated Spectrin Associated Protein 1) is probably a microtubule-binding protein that plays a role in the regulation of cell morphology and cytoskeletal organization. Through interaction with spectrin, CAMSAP1 may regulate neurite outgrowth and GO annotations related to this gene include microtubule binding and spectrin binding. The following gene GNG3 (G Protein Subunit Gamma 3) has been shown to have GTPase activity and G-protein coupled receptor binding activity from the GO annotations. Among its related pathways are GABAergic synapse and p75 NTR receptor-mediated signaling. All these four genes were significantly down-regulated in advanced aging-brain (GSE53890). Significantly lower expression of these genes was also validated in the aging-brain in other cohorts (GSE71620, GSE30272, and GSE11882, **Figure 5D**). These data suggested that PPP3CB, CAMSAP1, ACTR3B, and GNG3 might function as the novel candidate biomarkers for the normal brain aging.

### Hub Genes Were Significantly Down-Regulated in the Front Cortex From Aging Mices

**FIGURE 6:**
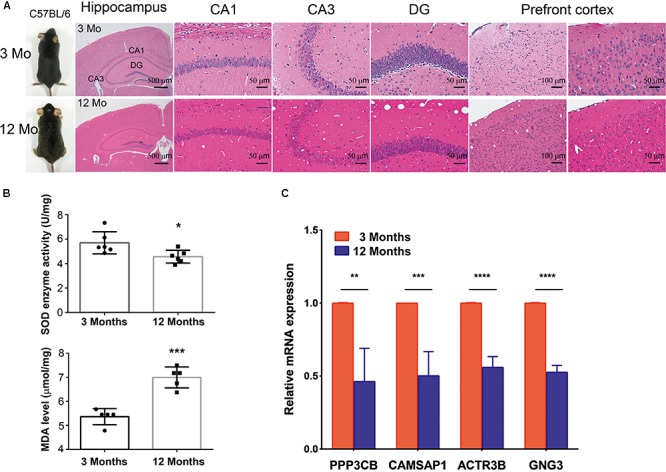
Expression of hub genes in different aging stage of C57BL/6J mice brain. **(A,B)** The representative HE staining of hippocampus and prefrontal cortex with different age stages were shown. The production of SOD and MDA in different age of prefrontal cortex was presented. **(C)** Quantification of hub genes was confirmed and presented. ^∗^*p* < 0.05, ^∗∗^*p* < 0.0, ^∗∗∗^*p* < 0.001, ^∗∗∗∗^*p* < 0.0001.

To further investigated whether hub genes expressed differentially across the progressive stages of brain aging, the 3-month-old and 12-month-old male C57BL/6 mices were used. HE staining of brain tissue with different age stages was shown to assess aging severity. The expression level of SOD and MDA were also tested in the 3-month-old and 12-month-old male C57BL/6 mices’ PFC (*n* = 6 in each group, **Figures 6A,B**), revealing that aged mices compared to young mices showed low level of SOD enzyme activity and high level of MDA. Then, to explore if hub genes were modified in the different stage of brain aging, we measured PPP3CB, CAMSAP1, ACTR3B, and GNG3 mRNA levels in extracts of PFC from young adult (3 months) and aged (12 months) individuals. Similarly, the mRNA level of PPP3CB, CAMSAP1, ACTR3B, and GNG3 were both remarkably down-regulated in the aging mice’s PFC, as verified by quantitative real time RT-PCR (qRT-PCR) (*n* = 3 in each group, **Figure 6C**). The data *in vivo* above indicates a rather close relationship between hub genes and normal brain aging progression.

### The Main Functional Organization of the Blue Module

**FIGURE 7:**
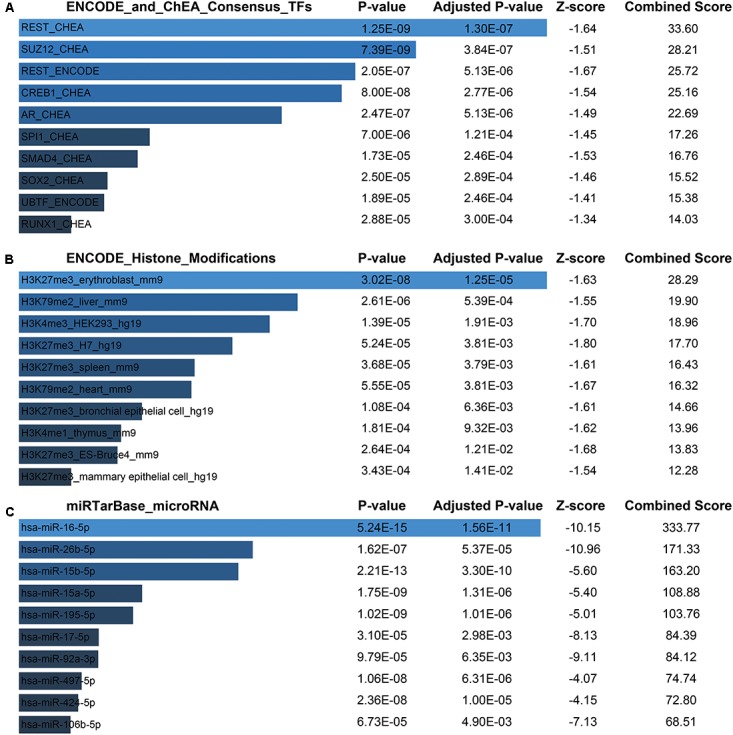
Potential factors regulating genes in blue module. **(A)** Transcription factors. **(B)** Histone modification markers. **(C)** Enrichment of associated microRNA.

Next, for a more intuitive depiction of interesting modules, the OmicShare tools, a free online platform for data analysis^4^, was used to re-annotated the functional relevance of blue and magenta module. With the cutoff set as *Q*-value < 0.05, synaptic vesicle cycle, cGMP-PKG, and dopaminergic synapse signaling pathway made up the main KEGG signaling pathways in blue module and the top of three oxidative phosphorylation, Huntington’s disease and Parkinson’s disease pathways constituted the main KEGG signaling pathways in magenta module, which were both depicted in bubble plots (**Supplementary File 2, Figure S3B**). For the blue module, the GO term of “transport” and “establishment of localization” were significantly enriched. The top enriched GO terms for magenta module were “mitochondrion organization” and “gene expression” (**Supplementary File 2, Figure S3A**). Moreover, there was a widespread consensus that co-expressed genes may be co-regulated by the common transcription factors (TFs), histone modification and microRNAs, so we performed a gene-set enrichment analysis by using ChEA, Encode, and TargetScan database (Lachmann et al., 2010; Mouse et al., 2012; Agarwal et al., 2015) for blue module. Thus, the top of significantly enriched TFs were observed for REST (RE1-Silencing Transcription factor), SUZ12 (SUZ12 polycomb repressive complex 2 subunit), CREB1 (CAMP Responsive Element Binding Protein 1), AR (androgen receptor), etc. (**Figure 7A** and **Supplementary File 1, Table S7**). Consistently, several studies showed that those TFs were functionally associated with brain aging. For instance, the elevated REST levels were closely related with increased longevity in aging humans by regulating a neuroprotective stress response during aging (Lu et al., 2014). For SUZ12, reports showed SUZ12 expression may regulate the transition from proliferation to cellular senescence (Overhoff et al., 2014). Specifically, in brain, the cyclic AMP responsive element binding protein1 (CREB1) TF was found to be involved in CREB signaling leading to cognitive deficits as observed in normal aging and neurodegenerative diseases by regulating specific genes (Paramanik and Thakur, 2013). Most recently, study showed that CREB1 was activated by nutrient deprivation in adult neurons and mediated the improved cognitive, electrophysiological, and pro-survival effects of low calorie intake (Fusco et al., 2016). Meanwhile, in the rat liver, AR expression might predict liver aging (Song et al., 1991; Supakar et al., 1993). As we known, dietary calorie restriction could retard age-related diseases and extends the invertebrate and vertebrate lifespan; interestingly, reversed loss of AR expression and restored androgen sensitivity in the aging liver were also observed during dietary calorie restriction (Song et al., 1991; Roy et al., 1996). Meanwhile, H3 lysine 27 trimethylation (H3K27me3) got a strongly enrichment for most of the genes in blue module (**Figure 7B** and **Supplementary File 1, Table S8**). It had shown that H3K27me3 was remodeled during early development, and H3K27me3 was a repressive epigenetic mark that changed dynamically during pre-implantation development in mice, bovine and pig embryos (Bogliotti and Ross, 2012). Finally, the most enriched miRNAs were observed for hsa-miR-16-5p, hsa-miR-26b-5p, hsa-miR-15b-5p, hsa-miR-15a-5p (**Figure 7C** and **Supplementary File 1, Table S9**). Study had indicated that the miR-15 family (miR-15a, miR-15b) was significantly down-regulated in the stress-induced premature senescence (SIPS) of the human diploid fibroblast (HDF) and human trabecular meshwork (HTM) cells (Li et al., 2009). In addition, miR-15b was a negative regulator of stress-induced SIRT4 expression thereby counteracting senescence associated mitochondrial dysfunction and regulating the senescence-associated secretory phenotype (SASP) and possibly organ aging, such as photoaging of human skin (Lang et al., 2016). Another study had shown forced expression of miR-16 could enhance p21 expression via down-regulation of the polycomb group protein Bmi1, thereby inducing cellular senescence (Kitadate et al., 2016). However, There were still no evidences whether the expression of hsa-miR-16-5p, hsa-miR-26b-5p, hsa-miR-15b-5p, hsa-miR-15a-5p changed with human brain aging.

## Discussion

The declining of cognitive function during aging has emerged as one of the major medical challenges of the 21st century. Earlier studies have demonstrated that neuronal loss is an integral feature of the aging brain. More recently, it is becoming clear that neuronal cell number is largely preserved and keeps their cognitive function relatively intact in the neocortex and hippocampus of the aging human brain, declining only in the setting of neurodegenerative disease (Gomez-Isla et al., 1996; Peters et al., 1998; Yankner et al., 2008; Lu et al., 2014). So, investigating how genes jointly preserve neurons and cognitive function relatively well during human brain aging is important, yet challenging. Recently increasing studies focus on high-throughput sequencing approach to investigated the regulation of normal brain aging and WGCNA is characterized effectively and systematically to find modules and gene signatures highly related with the clinical trait, such as the trait of brain aging. In our study, the modules and hub genes identified here are biologically rational. By using this analytical approach, 19 brain aging related modules were identified from the 41 human brain aging samples by reducing the complexity of the expression profiles. Among them, six modules were found to be significantly associated with brain aging progression. Moreover, the conservation of six modules among different datasets were also extensively studied. Further, we confirmed that the blue and magenta modules might serve as the main driver of brain aging based on the WGCNA meta-module, and further through the network feature (GS, MM, and K.in) analysis. Meanwhile, the enrichment of GO terms or pathway for blue and magenta module was also highly concordant. In particular, pathway analysis of these modules revealed that synaptic vesicle cycle, cGMP-PKG signaling pathway and oxidative phosphorylation were the top core gene sets of the blue and magenta module in human brain aging. The effect of oxidative phosphorylation on brain aging had been supported by lots of researches which report the aging of mammalian brain was associated with a continuous decrease of the capacity to produce ATP by oxidative phosphorylation (Ferrandiz et al., 1994; Boveris and Navarro, 2008; Chakrabarti et al., 2011). Correspondingly, reports showed that cGMP-PKG signaling pathway might have a relatively relationship with the procedures of brain aging. For example, study reported that the effect of aging (4-, 12-, and 24-month-old animals) on the glutamate-cyclic GMP-PKG could modulate alpha1, alpha(2/3)-Na, K-ATPase activity in rat cerebellum and stimulate the glutamate-cyclic GMP–PKG pathway at different levels by progressively decreased of cyclic GMP levels, PKG basal activity and alpha(2/3)-Na, K-ATPase activity (Scavone et al., 2005). In addition, we found synaptic vesicle cycle signaling pathway was highly associated with brain aging. However, few studies had reported synaptic vesicle cycle could affect the normal brain aging or neurodegenerative diseases. Thus, whether synaptic vesicle cycle signaling pathway was related to aging requires further validation. Besides, to make full use of blue module in the development of efficient anti-brain aging strategies, small compounds derived from the Library of Integrated Network-based Cellular Signatures (LINCS) L1000 platform (Vempati et al., 2014; Lou et al., 2017) affecting the blue module’s gene expression was shown in **Supplementary File 1, Tables S13–S15** and **Supplementary File 2, Figure S5**. And these candidate compounds might offer new drug interfere strategies in the development of brain aging. Next, novel potential biomarkers including PPP3CB, CAMSAP1, ACTR3B, and MFSD4A were confirmed in blue module, after extensive cross-validation. Interestingly, the co-expression mode of genes in blue module and its regulators (TFs and epigenetic markers), which might regulate the circuit during normal human brain aging progression, were noteworthy. Our study also has some limitations. First, there are a number of genes in blue module, we only select the top of 125 interesting genes in the blue module based on the indexes of GS, as well as MM and K.in, which may be biased in investigating the hub genes regulating the brain aging to some extent. Second, even though the hub genes in blue module are implicated in aging as validated by the mRNA expression of different GEO datasets and aging mices, as well as reported by some literature annotations, there are still a lot of experiments needed to validate these discovery clues. Recently studies suggest that almost all aged brains show characteristic changes that are linked to neurodegeneration. Therefore, this raises the question whether these characteristic changes represent lesser aspects of brain aging that do not considerably affect function or whether they are the harbingers of neurodegenerative diseases (Wyss-Coray, 2016). However, in our study, the postmortem brain tissue samples were neuropathologically normal and non-demented from the NCBI Gene Expression Omnibus. And only the transcriptomic profile from cognitively normal individuals at their certain ages were studied in WGCNA analysis. Besides, to test the stability of the indicated modules, we retrieved four datasets, which was also relevant to normal brain aging. Results showed that the anti-aging modules (blue, magenta, darkolivegreen) were preserved stably in GSE11882, GSE30272, GSE71620, and GSE1572 datasets (**Figure 3**). Taken together, this study generated a systematic and unbiased view of brain aging related modules and genes. In particular, blue module and genes regulating normal brain aging progression deserved further attention, which might be exploited as a novel biomarker for the evaluation of anti-aging interventions and highlight potential new targets for the prevention or treatment of age-associated brain disorders such as Alzheimer’s disease.

## Author Contributions

The specific work of each author in this study was as follows: HL: perception and final approval of the version to be published. YH: participation in the whole work, drafting of the article, and data analysis, JP, YX, and JW: feeding the animals and responsible for the brain samples collection. XM and QG: RT-PCR data acquisition and assessment.

## Conflict of Interest Statement

The authors declare that the research was conducted in the absence of any commercial or financial relationships that could be construed as a potential conflict of interest. The reviewer ML and handling Editor declared their shared affiliation at the time of review.

## Footnotes

1 https://www.ncbi.nlm.nih.gov/gds

2 http://amp.pharm.mssm.edu/Enrichr

3 http://david.abcc.ncifcrf.gov/

4 www.omicshare.com/tools
