# Supplementary material for: Gene Expression Analysis Reveals Novel Gene Signatures Between Young and Old Adults in Human Prefrontal Cortex
Source: Front Aging Neurosci. 2018 Aug 27;10:259. doi: 10.3389/fnagi.2018.00259 (PMC6119720; doi:10.3389/fnagi.2018.00259)
Supplement: Supplementary file 2 [file Data_Sheet_2.zip › Supplementary materials/Supplementary File 2.docx]

**Gene Expression Analysis Reveals Novel Gene Signatures and Candidate Molecular Mechanisms Between Young and Old Adults in Human Prefrontal Cortex**

*Yang Hu ^1,2,3^, Junping Pan ^1^, Yirong Xin ^1^, Xiangnan Mi ^1^, Jiahui Wang ^1^, Qin Gao ^1^,* *Huanmin Luo ^1,3*^*

*^1^ Department of Pharmacology, School of Medicine, Jinan University, Guangzhou, PR China, ^2^ Discipline of Pathology and Pathophysiology, School of Medicine, Jinan University, Guangzhou, PR China, ^3^ Institute of Brain Sciences, Jinan University, Guangzhou, PR China*

*^*^Correspondence: Dr. Huan-Min Luo: tlhm@jnu.edu.cn*


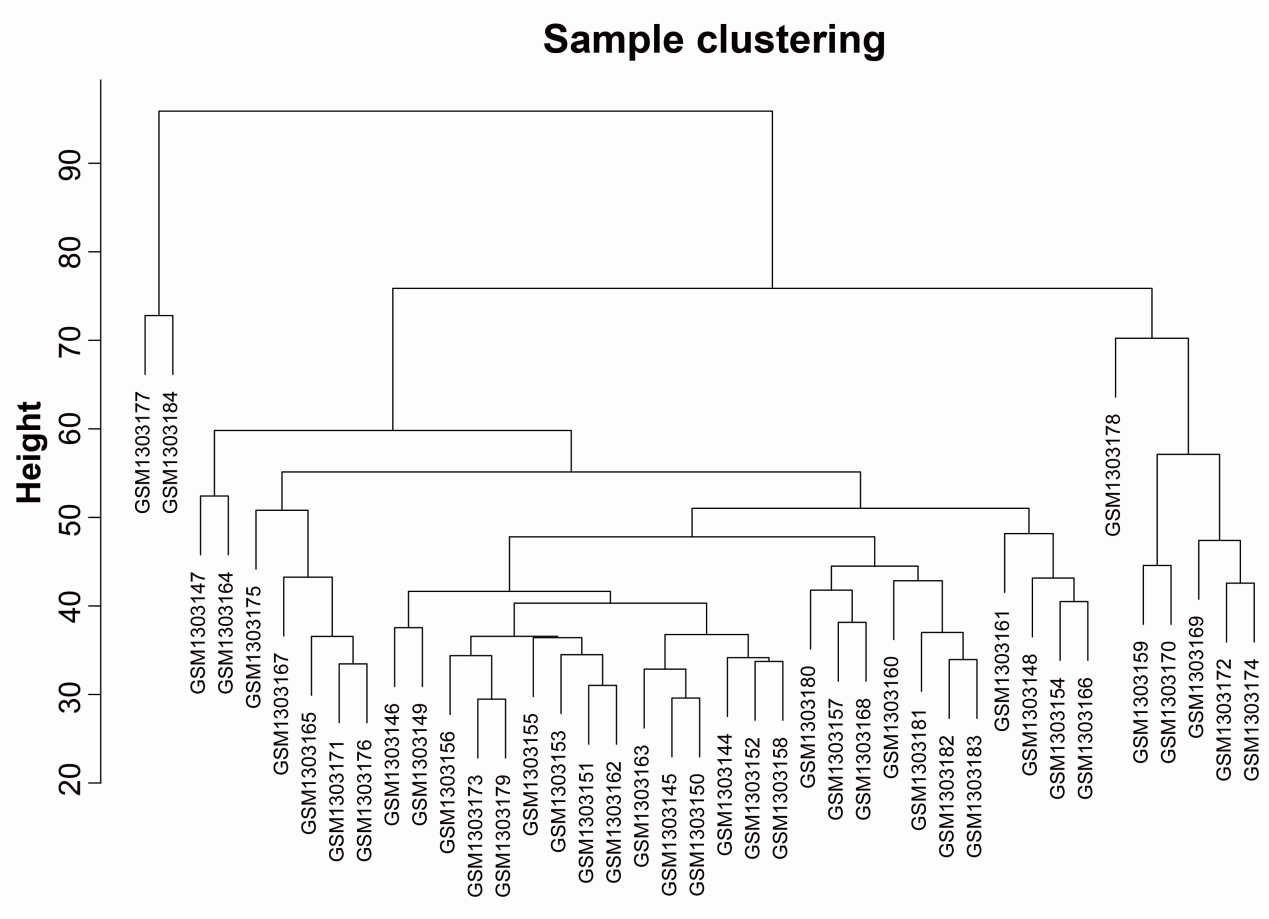


**Supplementary Figure S1. Hierarchical average linkage clustering to detect outlier samples.**


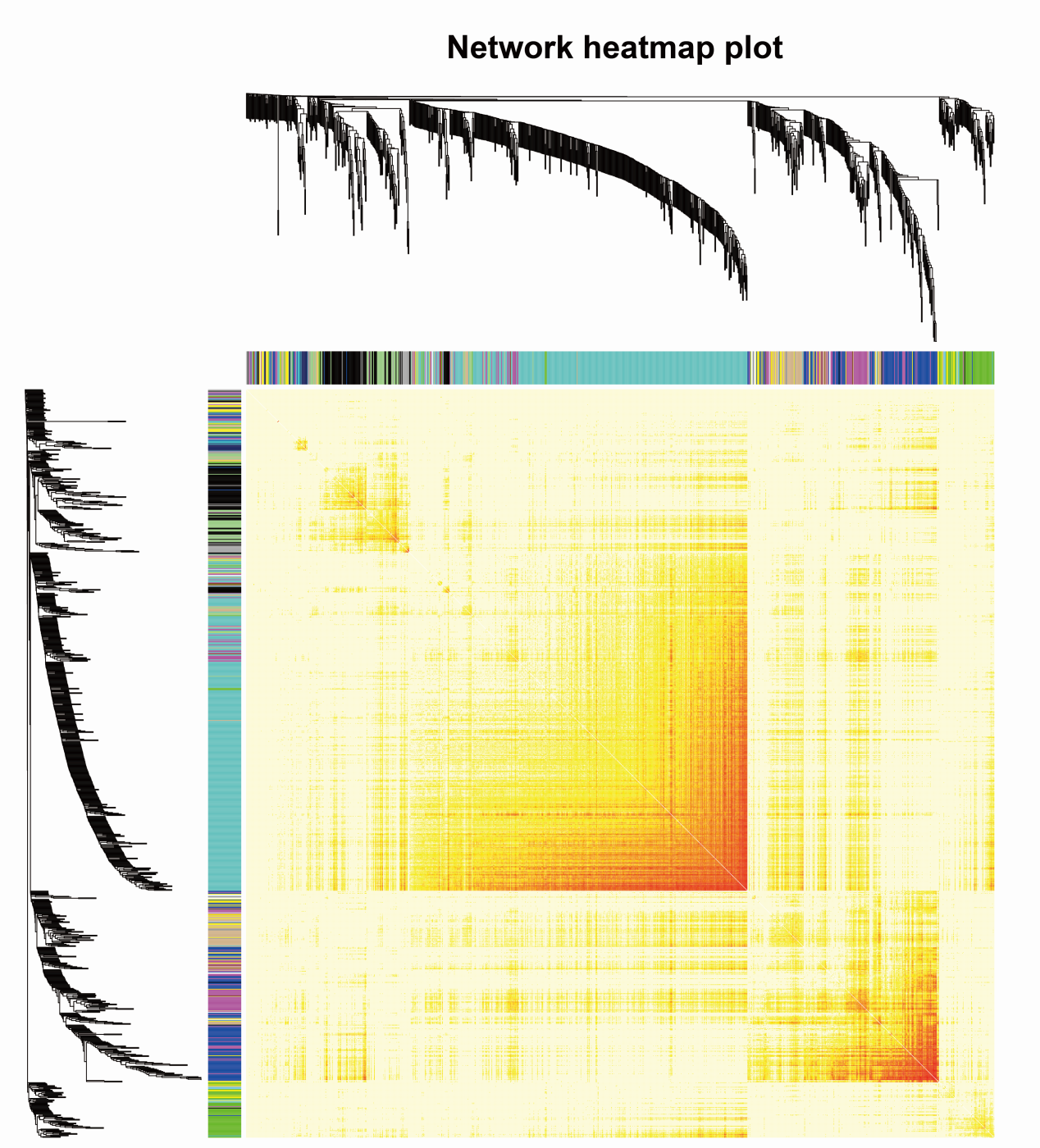


**Supplementary Figure S2. Network heatmap plot. Branch in the hierarchical clustering dendrograms correspond to each module.** Color-coded module membership is showed in the color bars below and to the right of the dendrograms. In the heatmap, the progressively more saturated yellow and red colors indicate the high co-expression interconnectedness. Modules correspond to highly interconnected genes blocks. Genes with high intramodular connectivity are located at the tip of the module branches since they exhibit the highest interconnectedness with the rest of the genes in the module.


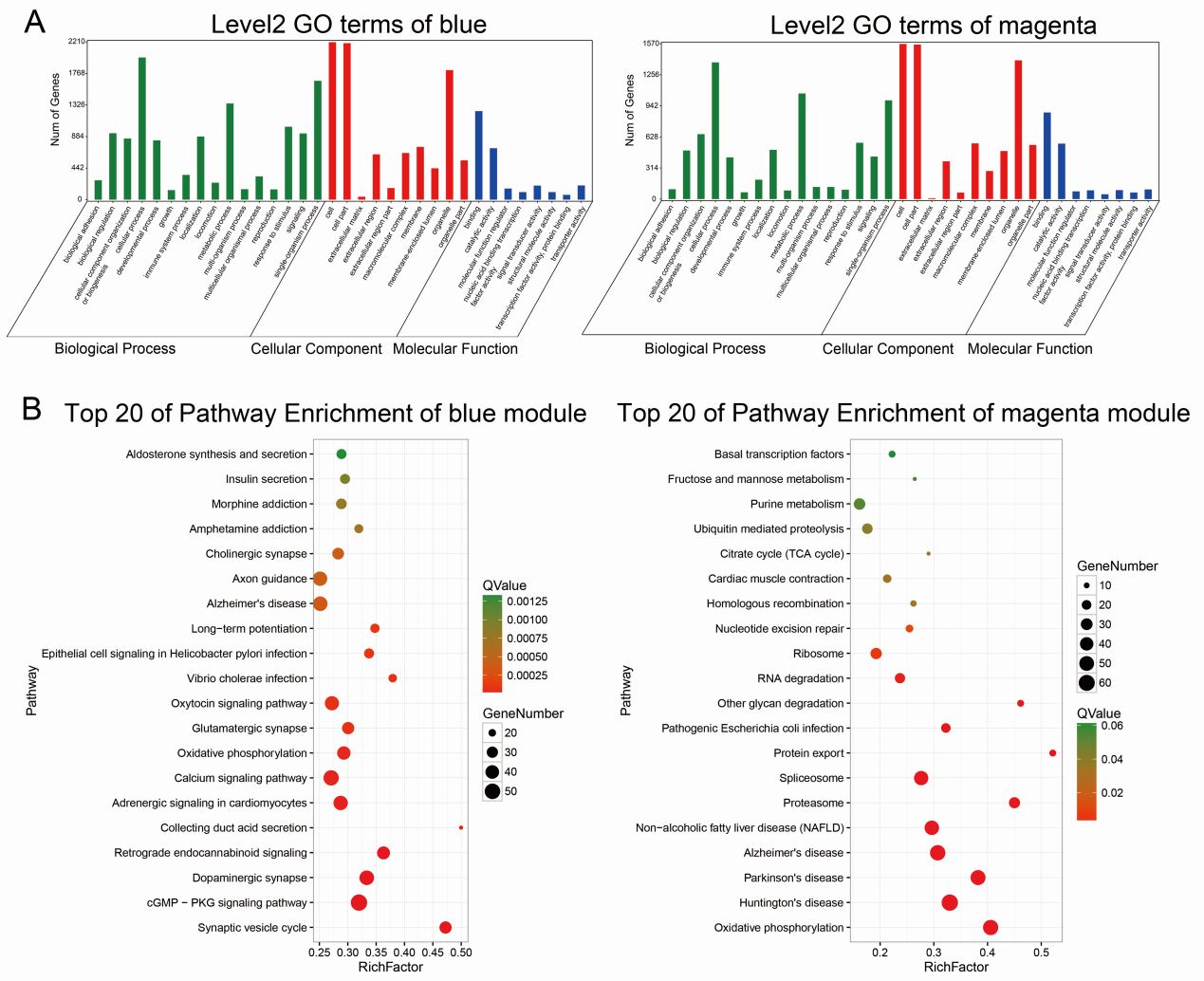


**Supplementary Figure S3. GO and KEGG enrichment analysis for blue and magenta modules.** (A) GO enrichment analysis in the blue module and the magenta module. (B) The top 20 KEGG pathways in the blue module and the magenta module.


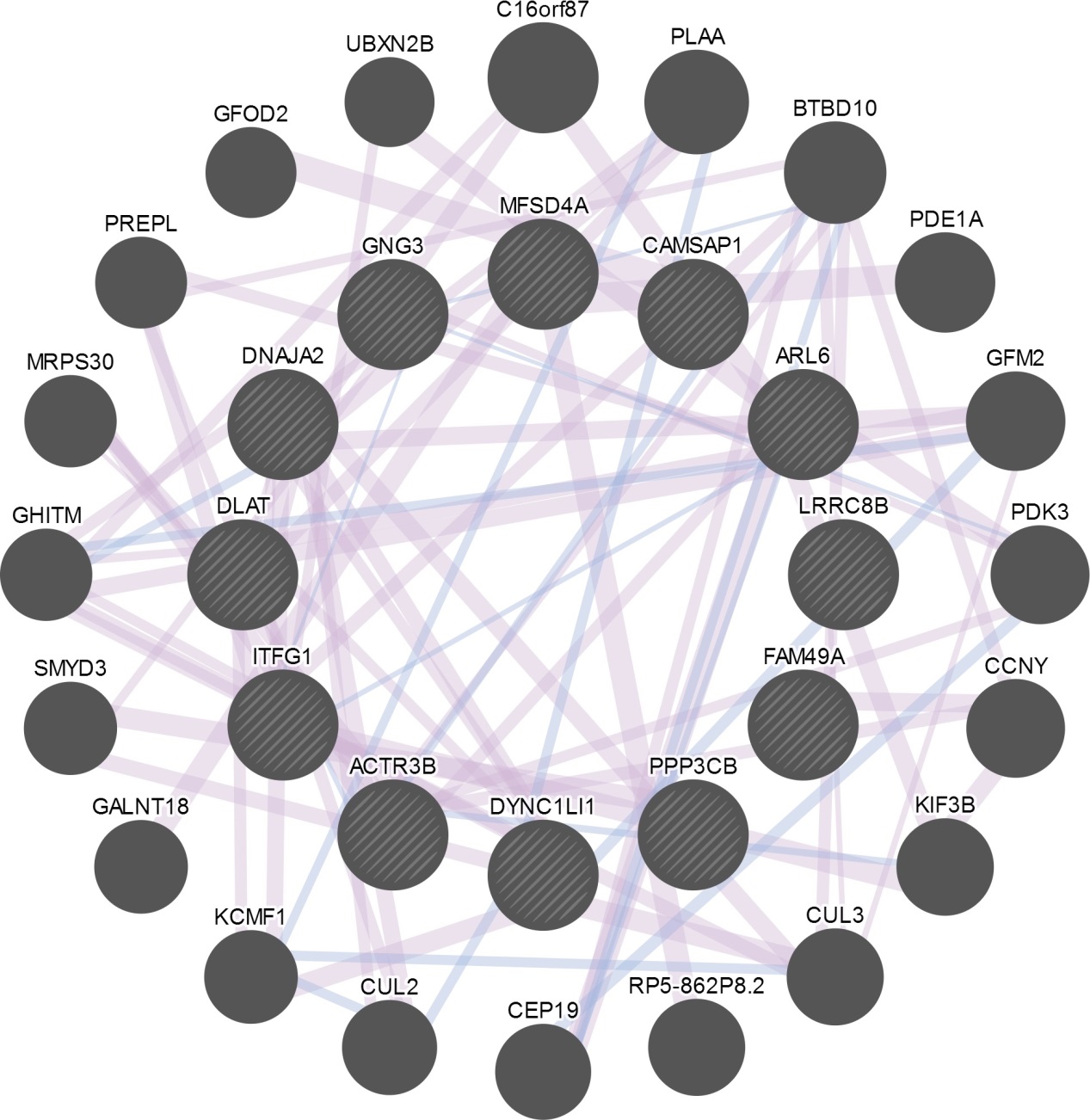


**Supplementary Figure S4. Topology of Key genes including 4 hub genes were well preserved among the networks produced by by Genemania.**


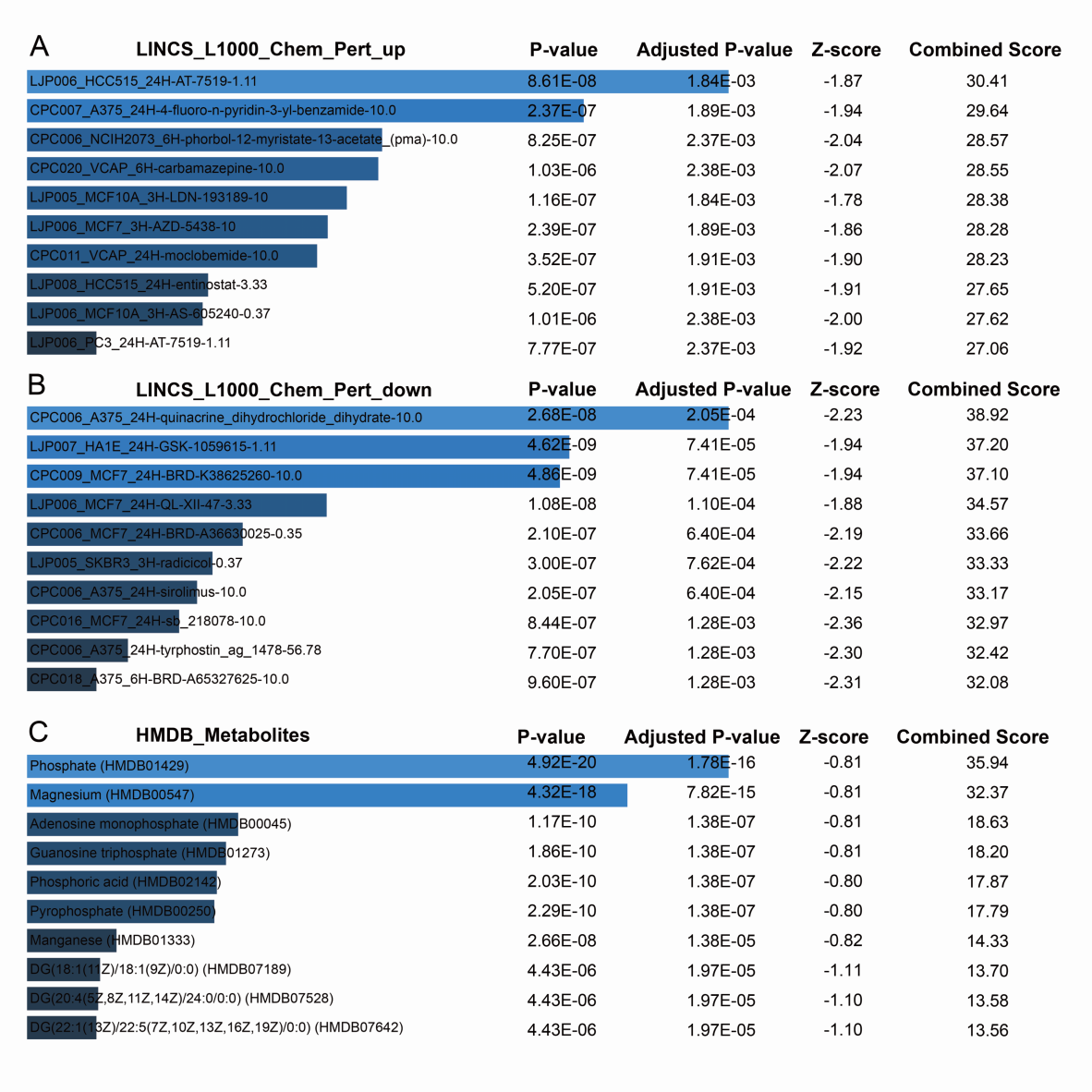


**Supplementary Figure S5. Enrichment analysis of module blue against ChEA database.**

**Supplementary Table S1. Datasets used in this study**

| **Accession** | **Platform** | **Title** | **Samples** | **Brief information** |
| --- | --- | --- | --- | --- |
| GSE53890 | GPL570:[HG-U133_Plus_2] Affymetrix Human Genome U133 Plus 2.0 Array | Age effect on normal adult brain: frontal cortical region | 41 | adult human brain samples from frontal cortical regions, including samples from 12 young (<40yr), 9 middle aged (40-70yr), 16 normal aged (70-94yr), and 4 extremely aged (95-106yr). |
| GSE1572 | GPL8300:[HG_U95Av2] Affymetrix Human Genome U95 Version 2 Array | Aging brain: frontal cortex expression profiles at various ages | 30 | the human frontal cortex from individuals ranging from 26 to 106 years of age. The postmortem brain tissue samples used in this study were neuropathologically normal for age, and were derived from non-demented individuals. |
| GSE71620 | GPL11532:[HuGene-1_1-st] Affymetrix Human Gene 1.1 ST Array | The effects of aging on circadian patterns of gene expression in the human prefrontal cortex | 146 | a total of 146 individuals with the following characteristics were analyzed in this study: mean (range) age of 50.7 (16-96) years,in the human prefrontal cortex (Brodmann’s areas (BA) 11 and 47). |
| GSE30272 | GPL4611:Illumina Human 49K Oligo array (HEEBO-7 set) | Temporal Dynamics and Genetic Control of Transcription in the Human Prefrontal Cortex | 269 | 269 human prefrontal cortex samples ranging from fetal development (negative ages) through aging (80 years). |
| GSE11882 | GPL570:[HG-U133_Plus_2] Affymetrix Human Genome U133 Plus 2.0 Array | Gene expression changes in the course of normal brain aging are sexually dimorphic | 173 | superior frontal gyrus (SG) across the lifespan of individuals from 20-99 years old. |

**Supplementary Table S2. The trait of samples in GSE53890**

| **Number** | **Samples** | **Age** | **Sex** |
| --- | --- | --- | --- |
| 1 | GSM1303144 | 24 | Male |
| 2 | GSM1303147 | 26 | Male_2 |
| 3 | GSM1303148 | 26 | Male |
| 4 | GSM1303151 | 29 | Male |
| 5 | GSM1303155 | 37 | Male |
| 6 | GSM1303145 | 25 | Female |
| 7 | GSM1303146 | 25 | Female_2 |
| 8 | GSM1303149 | 27 | Female |
| 9 | GSM1303150 | 29 | Female |
| 10 | GSM1303152 | 33 | Female |
| 11 | GSM1303153 | 34 | Female |
| 12 | GSM1303154 | 36 | Female |
| 13 | GSM1303156 | 40 | Male |
| 14 | GSM1303159 | 45 | Male |
| 15 | GSM1303161 | 51 | Male |
| 16 | GSM1303162 | 52 | Male |
| 17 | GSM1303164 | 66 | Male |
| 18 | GSM1303157 | 44 | Female_2 |
| 19 | GSM1303158 | 44 | Female |
| 20 | GSM1303160 | 48 | Female |
| 21 | GSM1303163 | 61 | Female |
| 22 | GSM1303165 | 70 | Male |
| 23 | GSM1303167 | 77 | Male |
| 24 | GSM1303169 | 82 | Male |
| 25 | GSM1303170 | 84 | Male |
| 26 | GSM1303172 | 86 | Male |
| 27 | GSM1303174 | 89 | Male |
| 28 | GSM1303175 | 90 | Male |
| 29 | GSM1303177 | 91 | Male |
| 30 | GSM1303178 | 92 | Male |
| 31 | GSM1303166 | 71 | Female |
| 32 | GSM1303168 | 80.5 | Female |
| 33 | GSM1303171 | 86 | Female |
| 34 | GSM1303173 | 87 | Female |
| 35 | GSM1303176 | 91 | Female |
| 36 | GSM1303179 | 93 | Female |
| 37 | GSM1303180 | 94 | Female |
| 38 | GSM1303182 | 104 | Male |
| 39 | GSM1303181 | 103 | Female |
| 40 | GSM1303183 | 105 | Female |
| 41 | GSM1303184 | 106 | Female |
